# Protected-Area Boundaries as Filters of Plant Invasions

**DOI:** 10.1111/j.1523-1739.2010.01617.x

**Published:** 2011-04

**Authors:** Llewellyn C Foxcroft, Vojtěch JaroŠÍK, Petr Pyšek, David M Richardson, Mathieu Rouget

**Affiliations:** * Conservation Services, South African National ParksSkukuza 1350, South Africa email llewellynf@sanparks.org; † Centre for Invasion Biology, Department of Botany and Zoology, Stellenbosch UniversityPrivate Bag X1, Matieland 7602, South Africa; ‡ Department of Ecology, Faculty of Sciences, Charles University in PragueViničná 7, CZ 128 44 Prague 2, Czech Republic; § Institute of Botany, Department of Invasion Ecology, Academy of Sciences of the Czech RepublicPrůhonice, CZ 252 43, Czech Republic; ** Department of Plant Science, University of PretoriaPretoria 0002, South Africa

**Keywords:** barriers to invasion, Kruger National Park, non-native invasive species, overland water flow, protected-area boundary, barreras contra invasión, especies invasoras no nativas, flujo de agua superficial, límite de área protegida, Parque Nacional Kruger

## Abstract

**Abstract:**

Human land uses surrounding protected areas provide propagules for colonization of these areas by non-native species, and corridors between protected-area networks and drainage systems of rivers provide pathways for long-distance dispersal of non-native species. Nevertheless, the influence of protected-area boundaries on colonization of protected areas by invasive non-native species is unknown. We drew on a spatially explicit data set of more than 27,000 non-native plant presence records for South Africa's Kruger National Park to examine the role of boundaries in preventing colonization of protected areas by non-native species. The number of records of non-native invasive plants declined rapidly beyond 1500 m inside the park; thus, we believe that the park boundary limited the spread of non-native plants. The number of non-native invasive plants inside the park was a function of the amount of water runoff, density of major roads, and the presence of natural vegetation outside the park. Of the types of human-induced disturbance, only the density of major roads outside the protected area significantly increased the number of non-native plant records. Our findings suggest that the probability of incursion of invasive plants into protected areas can be quantified reliably.

## Abstract (translated)

Límites de Áreas Protegidas como Filtros para la Invasión de Plantas

**Resumen:**

Los usos de suelo alrededor de áreas protegidas proporcionan propágulos para la colonización de estas áreas por especies no nativas, y los corredores entre las redes de áreas protegidas y los sistema fluviales proporcionan medios para la dispersión a larga distancia de especies no nativas. Sin embargo, se desconoce la influencia de los límites del área protegida sobre la colonización de áreas protegidas por especies invasoras no nativas. Utilizamos un conjunto de datos espacialmente explícitos con más de 27,000 registros de la presencia de especies de plantas no nativas en el Parque Nacional Kruger en Sudáfrica para examinar el papel de los límites en la prevención de la colonización de áreas protegidas por especies no nativas. El número de registros de plantas invasoras no nativas disminuyó rápidamente más allá de 1500 m dentro del parque; por lo tanto, consideramos que el límite del parque impidió la dispersión de plantas no nativas. El número de plantas invasoras no nativas dentro del parque fue una función de la escorrentía de agua, la densidad de caminos principales y la presencia de vegetación natural fuera del parque. De los tipos de perturbación humana inducida, solo la densidad de caminos principales fuera del área protegida incrementó el número de registros de plantas no nativas. Nuestros hallazgos sugieren que la probabilidad de incursión de plantas invasoras hacia áreas protegidas puede ser cuantificada confiablemente.

## Introduction

Many anthropogenic threats to native species of plants and animals are not removed by establishing formal protected areas. Theoretically, the probability that protected areas will meet their goals is improved by establishing networks of protected areas and by increasing connectivity through the creation of corridors (Foxcroft et al. 2007; Gaston et al. 2008). Such strategies, however, do little to prevent non-native invasive species from colonizing protected areas (e.g., Lambdon et al. 2008; Pyšek et al. 2008; Hulme et al. 2009). Some types of landscape features may exacerbate such colonizations; for example, river networks can facilitate colonization of non-native plants (Pyšek & Prach 1993; Margules & Sarkar 2007; Richardson et al. 2007). Protected-area management strategies that address colonization of non-native species generally focus on early detection and eradication, and action is applied only to species that are likely to have greatest negative effects on ecosystem functions. Establishment of buffer zones around protected areas is often included in these strategies. Although some researchers have addressed non-native species colonization at the interface between protected areas and human-dominated systems (Pyšek et al. 2003; Alston & Richardson 2006), no one has addressed the distance of non-native species’ incursions into protected areas or what would constitute an effective and sustainable width of buffer to reduce incursions.

**Figure 1:**
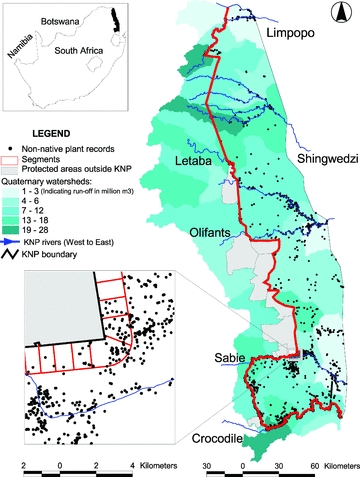
Mean annual river run off in Kruger National Park (KNP), pattern of non-native plant records relative to KNP boundary and segments (lower left inset), and location of KNP within South Africa (upper left inset).

A data set on distributions of non-native plant species and land use in and around South Africa's Kruger National Park (KNP) allowed us to conduct a detailed analysis of the permeability of protected area boundaries. KNP, in northeastern South Africa (Fig. 1), was founded in 1898 and covers an area of approximately 20,000 km^2^. More than 370 non-native species have been recorded in the park (Foxcroft & Freitag-Ronaldson 2007). In response, KNP managers have initiated programs aimed at preventing and mitigating colonization of the park by non-native species (Foxcroft & Downey 2008; Koenig 2009), and detailed data on the distribution of these species have been collected over 4 years (Foxcroft et al. 2009).

We explored the extent to which park boundaries act as a barrier to the influx of non-native plant species from outside to inside the park. We also examined how particular characteristics of the surrounding landscape, such as land use and presence of features thought to promote dispersal of non-native plants, affect the number and location of records of non-native plants inside the park. Thus, we sought to identify factors that could be used to explain which areas are more likely to be invaded and how far invasions of non-native species extend into the park. Such insights are needed to explain invasion patterns and elucidate management options for this and other protected areas.

## Methods

### Study Area

We assessed the relation between the western and southern boundaries of KNP and colonization of the park by non-native invasive plants. We excluded the northern (Limpopo River) and eastern (border with Mozambique) boundaries from our analyses (Fig. 1). We based this delimitation on the assumption that propagules of non-native species arrive mainly from the western side of the KNP because all rivers flowing through the park flow from west to east and tourist entrance gates are primarily along the western and southern boundaries. Also, data from outside South Africa (Mozambique in the east and Zimbabwe in the north) are not as spatially comprehensive as data from South Africa. Additionally, the Limpopo River has an extensive drainage basin of which the KNP is only 4%; including this edge would thus distort the effects of water runoff on the potential colonization of non-native plants.

### Data Set

Approximately 120 field rangers collect data during their daily patrols with a hand-held personal computer and customized software (CyberTracker; MacFadyen 2005; Foxcroft et al. 2009). Rangers record data opportunistically as they move through an area. Apart from the presence of non-native plants, rangers also recorded observations of animals, carcasses, and tracks and water availability (Foxcroft et al. 2009), which we used as non-native plant absence points. We considered a non-native species to be present at a given point if a ranger recorded its occurrence and absent if a ranger recorded a visit to the point but did not detect a plant or record any other observation. Absence records were assumed to be accurate on the basis of the assumption that if a non-native plant was present at the same point as another observation, its presence would also have been recorded. We believe that this assumption is justified because we considered only the most abundant and conspicuous non-native species that trained rangers could recognize reliably: erect pricklypear (*Opuntia stricta*), lantana (*Lantana camara*), triffid weed (*Chromolaena odorata*), and parthenium (*Parthenium hysterophorus*) (Foxcroft et al. 2009).

The data set is spatially explicit, covers all of KNP (Foxcroft et al. 2009), and includes >27,000 presence records and >2 million absence records collected between 2004 and 2007. Along the western and southern park boundaries we delineated 638 contiguous, 1 km wide segments that extended into the park 1.5 km (Fig. 1). All presence and absence records within the segments were used in the analyses.

### Nonnative Species

We extracted numbers of non-native records and proportions of non-native records among all records to account for a possible effect of sampling intensity by rangers. We determined the perpendicular distances of all non-native plant records (hereafter non-native records) and the proportions of non-native records among all records from the park boundary. We summed the distances within 100-m increments along a perpendicular line that ran from the boundary across the entire park (maximum distance was 52,000 m). Proportions of non-native records were calculated as the number of non-native records divided by the total number of non-native presence and absence records in the 100-m increments from the boundary up to a distance of 1700 m because thereafter the segments overlapped.

### Environmental Data

We examined the effects of several environmental variables on the presence of non-native plants in KNP. Inside the park, we summed the presence of roads, camps, gates, rivers, and the dominant type of vegetation within each segment along the boundary (Fig. 1) (Supporting Information). Outside the park, starting at the boundary and extending away from the park, we determined the density of major (national) roads and all (including secondary and gravel) roads, land use (within 1, 5, 10, and 50-km radii outside the park boundary [Supporting information]), mean annual water runoff, presence of protected area, and primary productivity of vegetation, expressed as either continuous or binary variables (Supporting Information).

### Statistical Analyses

We plotted the numbers and proportions of non-native records against the distance from the boundary, and analyzed with Kendall's rank correlation whether the occurrence of non-native plants decreased monotonically as distance from the KNP boundary increased (Legendre & Legendre 1998). To determine whether there was a threshold distance at which the proportion of non-native records became constant, we used a locally weighted, scatterplot smoothing regression model (LOWESS) to assess a region of effective neutrality (Trexler & Travis 1993), defined as the distance from the KNP boundary at which there was no relation between the distance and the number of non-native records (Cleveland 1979; Chambers et al. 1983).

We used a binary classification tree (Breiman et al. 1984; De'ath & Fabricius 2000) to analyze the presence and absence of non-native species in the segments as a function of environmental characteristics measured within and outside KNP. In the classification trees the data set was subdivided into homogenous groups on the basis of a series of hierarchical splits. The classification accuracy of each split in the tree was expressed by its improvement score (i.e., the overall number of misclassifications at each node). High improvement scores corresponded to splits of high quality. The quality of a tree was evaluated on the basis of the overall misclassification rate by comparing the misclassification rate of the optimal tree with 50% misclassification rate of the null model (De'ath & Fabricius 2000) and using cross-validated samples (Steinberg & Colla 1995) based on values of sensitivity (the ability of the model to predict that a non-native species is present when it is) and specificity (the ability of the model to predict that a non-native species is not present when it is not) (Bourg et al. 2005).

The environmental variables identified by the optimal classification tree as explaining the greatest variance in number of non-native records were not collinear when checked by calculating tolerance values (following Quinn & Keough, 2002). Thus, we used them as explanatory variables in a logistic regression with binomial errors and logit link function (Quinn & Keough 2002) in which the presence or absence of non-native species in each of the 638 segments was the response variable. Details of all analyses are described in Supporting Information.

## Results

**Figure 2:**
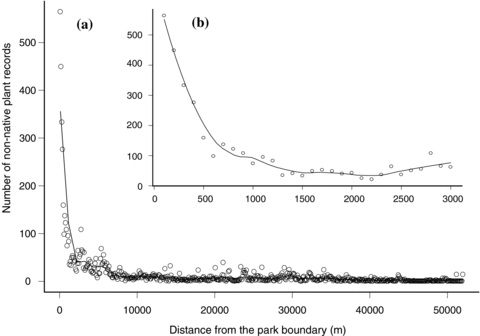
Number of presence records of non-native plants relative to the distance from the boundary of Kruger National Park toward the interior of the park: (a) 52,000 m and (b) 3000 m. Fitted curves are LOWESS regression models on square-root number of records (backtransformed for visualization) with no patterns of residuals. In (a) span of smoothing = 0.1; equivalent number of parameters (ENP) in curvilinear regression = 29.8; explained variance *r*^2^= 0.82; model chosen by deletion test (*F* =1.16; ENP = 313.2, 29.8; *p* =0.13) against starting model with span = 0.01 and *r*^2^= 0.94. In (b) span of smoothing = 0.5; ENP = 6.4; *r*^2^= 0.96; model chosen by deletion test (*F* =2.37; ENP = 15.1, 6.4; *p* =0.07) against starting model with span = 0.2 and *r*^2^= 0.99. The spans of smoothing describe local sensitivity in iterations of LOWESS models (Supporting Information).

Inside the park, the number of non-native records decreased monotonically as the distance from the KNP boundary increased (Fig. 2a,b) (Kendall's rank correlation τ=−0.53; *z*=−18.00; *p* < 0.0001 for the number of non-native records). The proportion of non-native records also decreased monotonically as the distance from the park boundary increased (Kendall's rank correlation τ=−0.71; *z*=−3.95; *p*= 0.0001). The difference between the distance of non-native records and the proportion of records, up to 1700 m from the boundary, was only 1.5% of the explained variance, whereas 93.6% of the explained variance was the same for the two measures (Supporting Information). This indicates that sampling bias associated with use of number rather than proportion of non-native records was negligible. At approximately 1.5 km from the KNP boundary, the number of non-native records became constant (Fig. 2b).

**Figure 3:**
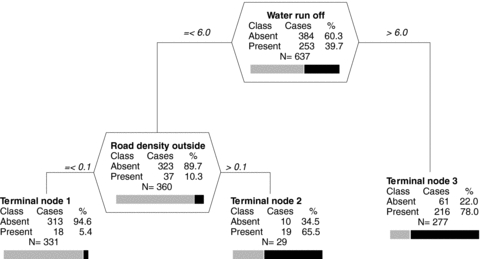
Results of classification tree analysis of the binary probability of the non-native species presence in Kruger National Park (KNP). Probability of presence was determined on the basis of water runoff in the park (mean annual runoff from the surrounding watershed; million m^3^) and road density adjacent to the park (density of major roads within 10-km radius outside the KNP boundary) (Supporting Information) (%, percentage of cases for each class; bars, representation of the percentage of absent [gray] and present [black]). The splitting variable name and split criterion are given for each node. Vertical depth of each node is proportional to its improvement value. Overall misclassification of the optimal tree was 14.0%, sensitivity 0.92 and specificity 0.81 (Supporting Information).

The mean annual water runoff (>6 million m^3^/annum) from the watershed outside the park explained the greatest proportion of variance in non-native records in a given segment. Segments with less than mean runoff were more likely to have non-native species present only in areas with high (>0.1 km/km^2^) road density within 10 km outside the park boundary (Fig. 3). The effects of water runoff and road density were complementary: high levels of runoff were associated with low road density and vice versa, but high values of both variables were not more strongly associated than either variable alone with an increase in number of non-native records (Supporting Information). In a model (Supporting Information) in which mean annual runoff was replaced by a categorical measure of runoff (none, no rivers intersected the segment; low, 2–10 million m^3^ċ quaternary watershed^−1^ċ year^−1^; medium, 10–15; high, 15–26), the number of non-native records in segments with no river present was associated again with the density of major roads within a 10-km radius outside the KNP boundary. If a segment contained a river, the presence of non-native plants was unlikely only in segments where natural vegetation (possibly grazed by livestock) comprised over 90% of the land use in the 5-km radius outside the KNP boundary and where roads were absent within the park (Supporting Information).

## Discussion

Our results suggest that the park boundary limits the spread of non-native plants into the KNP. We quantified the number of non-native records, which we assumed is a surrogate measure of boundary permeability, on the basis of a few environmental variables measured outside and within the park. These features are related to factors that facilitate the spread of non-native species, such as the presence of water courses (Pyšek & Prach 1993; Richardson et al. 2007), human activity (von der Lippe & Kowarik 2007), and road construction. Our statistical models had high explanatory power and we believe that they may be applicable to protected areas in general.

Of the two types of vectors of propagules of non-native plants, water runoff and road density, the association of water runoff with the number of non-native plants was stronger. Park managers have little control over the upper reaches of the rivers that flow through KNP. But it appears that there is a quantifiable threshold value of water runoff from surrounding areas below which invasion of non-native plants is less likely. This knowledge could be applied to prioritization of measures to control colonization of areas by non-native plants, such as targeting particular riparian areas for removal of non-native plants. The effects of water and road density on dispersal of non-native plants appeared to be similar but not synergistic. The number of non-native records was explained by environmental variables up to a distance of 10 km from the boundary; therefore, data on variables that explain significant variance in the presence of non-native invasive species can be collected relatively near the protected area.

These variables are associated with the spread of non-native plants into the park from surrounding landscapes. Within about 1.5 km into the park from the boundary, the number of non-native plants decreased sharply. This distance beyond which a rapid decline in abundance of non-native plants occurs is likely to differ among protected areas, ecosystems, and vegetation types, but we believe that such a threshold is likely to be present in all protected areas. This does not mean that park interiors cannot have high abundances of non-native plants species in places. Land use within a protected area can affect the spread of non-native plants (e.g., tourist camps and staff villages) and serve as sources of non-native species (Foxcroft 2001; Foxcroft et al. 2008). Further spread of non-native plants into KNP may be limited by placement of entrance gates where water runoff is low. Additionally, because the number of non-native plant records declined rapidly as the distance into the park increased, we believe that a buffer zone of undisturbed vegetation surrounding the entire park could slow the spread of non-native plant species.
