# Supplementary material for: Protected-Area Boundaries as Filters of Plant Invasions
Source: Conserv Biol. 2011 Apr;25(2):400–5. doi: 10.1111/j.1523-1739.2010.01617.x (PMC3085078; doi:10.1111/j.1523-1739.2010.01617.x)
Supplement: Supplementary file 1 [file cobi0025-0400-SD1.doc]

**Supporting Information**

**Appendix S1** Environmental variables used to explain the occurrence of non-native plants in the Kruger National Park (KNP).

|  | Category | Variable | Variable type |
| --- | --- | --- | --- |
| Outside KNP | ^a^Traffic | Density of major roads within 1, 5, 10, 50 km of boundary [km/km^2^] | Continuous |
|  |  | Density of all roads 1, 5, 10, 50 km [km/km^2^] | Continuous |
|  | ^b^Land use | % natural areas in 1, 5, 10, 50 km | Continuous |
|  |  | % cultivated areas in 1, 5, 10, 50 km | Continuous |
|  |  | % plantations in 10, 50 km | Continuous |
|  |  | % urban areas in 1, 5, 10, 50 km | Continuous |
|  |  | % degraded areas in 1, 5, 10, 50 km | Continuous |
|  | Protected areas | Protected areas adjacent KNP | Binary (present/absent) |
|  | ^c^Run-off from quaternary watershed | Mean annual runoff [million m^3^ / quaternary watershed / annum]  River runoff category [none, low, medium, high]. (*None* indicates that no main rivers intersected the segment) | Continuous  Categorical |
|  | Vegetation productivity | ^d^NDVI mean value | Continuous |
| Inside KNP | Human activities | ^a^Presence of major roads  Presence of roads  Presence of camps  Presence of gates | Binary (present/absent)  Binary (present/absent)  Binary (present/absent)  Binary (present/absent) |
|  | ^e^Rivers | Presence of main river  Presence of rivers | Binary (present/absent)  Binary (present/absent) |
|  | ^f^Vegetation type | Landscape unit (Gertenbach 1983) | Categorical |

Notes: Data was extracted for areas inside the park (summarized in 1 × 1.5 km segments on transects from the edge of the park towards its interior; Fig. 1), and outside the park (summarized for sections adjacent to each 1 km wide transect in a 1, 5, 10 and 50 km radius outside the park boundary).

^a^Major roads are defined as the main tourist tar roads in KNP

^b^Natural areas are untransformed landscapes (although probably grazed by livestock), cultivated areas are agricultural (crop) lands, plantations are commercial plantation forests, urban areas include towns and informal / rural settlements, degraded areas are areas transformed by erosion (gullies and bare soil), loss of plant cover, and other disturbances

^c^Refers to quaternary watersheds; Quaternary watersheds are nested subdivisions of primary, secondary and tertiary watersheds, where primary watersheds refer to the drainage areas of major rivers in the South African river classification system (van Wilgen *et al*. 2007). Run-off was only given for those segments for which a main river intersected it.

^d^Normalized Difference Vegetation Index; is a measure of the amount of green vegetation i.e. photosynthetically active material, and is used as a proxy for above-ground net primary production.

^e^Main rivers are defined as the seven main river systems (Limpopo, Luvuvhu, Shingwedzi, Letaba, Olifants, Sabie, Crocodile Rivers; Fig. 1) flowing into and through the KNP from west to east

^f^See Table S2.

References

Gertenbach, W. P. D. 1983. Landscapes of the Kruger National Park. Koedoe 26:9-121.

van Wilgen, B. W., J. L. Nel, and M. Rouget. 2007. Invasive alien plants and South African rivers: a proposed approach to the prioritization of control operations. Freshwater Biology 52:711–723.
