# Supplementary material for: Protected-Area Boundaries as Filters of Plant Invasions
Source: Conserv Biol. 2011 Apr;25(2):400–5. doi: 10.1111/j.1523-1739.2010.01617.x (PMC3085078; doi:10.1111/j.1523-1739.2010.01617.x)
Supplement: Supplementary file 2 [file cobi0025-0400-SD2.doc]

**Supporting Information**

**Appendix S2** Landscape units of Kruger National Park (KNP) used as an explanatory variable for non-native plant records in KNP.

| Landscape ID | Landscape^*^ | Description |
| --- | --- | --- |
| 1 | Lowveld Sour Bushveld of Pretoriuskop | Granitic plains with *Terminalia* *sericea* tree savanna |
| 2 | Malelane Mountain Bushveld | Granitic mountains with *Combretum* *apiculatum* bush savanna |
| 4 | Thickets of the Sabie & Crocodile Rivers | Granitic lowlands with *Acacia* *grandicornuta* tree savanna |
| 5 | Mixed *Combretum* / *Terminalia sericea* woodland | Granite plains with *Combretum* *zeyheri* or *Combretum* *apiculatum* bush savanna |
| 6 | Combretum / Colophospermum mopane woodland of Timbavati | Granite plains with *Colophospermum* *mopane* bush or tree savanna |
| 7 | Olifants River Rugged Veld | Granite plains with *Colophospermum* *mopane* bush or tree savanna |
| 8 | Phalaborwa Sandveld | Granitic plains with *Colophospermum* *mopane* bush savanna |
| 9 | *Colophospermum mopane* woodland / savanna on basic soil | Metalava with *Colophospermum* *mopane* tree savanna |
| 10 | Letaba River Rugged Veld | Metalava with *Colophospermum* *mopane* tree savanna |
| 11 | Tsende Sandveld | Granitic plains with *Colophospermum* *mopane* bush savanna |
| 12 | *Colophospermum mopane* / *Acacia nigrescens* savanna | Metalava plains with *Colophospermum* *mopane* tree savanna or Andesitic plains with *Combretum* *collinum* shrub savanna |
| 13 | *Acacia welwitschii* thickets on Karoo sediments | Karoo sediment plains with *Acacia* *welwitschii* tree savanna or with *Terminalia* *sericea* bush savanna |
| 16 | Punda Maria Sandveld on Cave Sandstone | Clarens sandstone hills with *Terminalia* *sericea* bush savanna or Soutpansberg group mountains with *Burkea* *africana* tree savanna |
| 17 | *Sclerocarya birrea* subspecies *caffra* /*Acacia* *nigrescens* savanna | Basaltic or gabbroic plains with *Sclerocarya* *birrea* tree savanna or *Acacia* *nigrescens* bush or shrub savanna |
| 19 | Thornveld on gabbro | Basaltic or gabbroic plains with *Sclerocarya* *birrea* tree savanna or *Acacia* *nigrescens* bush or shrub savanna |
| 24 | *Colophospermum mopane* shrubveld on gabbro | Basaltic or gabbroic plains with *Acacia* *nigrescens* bush savanna or *Colophospermum* *mopane* bush or shrub savanna |
| 25 | *Adansonia digitata* / *Colophospermum* *mopane* Rugged Veld | Basaltic or calcitic plains with *Colophospermum* *mopane* shrub savanna |
| 26 | *Colophospermum* *mopane* shrubveld on calcrete | Basaltic or calcitic plains with *Colophospermum* *mopane* shrub savanna |
| 28 | Limpopo / Luvuvhu Floodplains | Alluvial plains with *Faidherbia* *albida* or *Salvadora* *angustifolia* tree savanna |
| 29 | Lebombo South | Basaltic plains or rhyolite mountains with *Combretum* *apiculatum* or *Colophospermum* mopane bush savanna |
| 33 | *Pterocarpus rotundifolius* / *Combretum* *collinum* woodland | Metalava plains with *Colophospermum* *mopane* tree savanna or Andesitic plains with *Combretum* *collinum* shrub savanna |
| 34 | Punda Maria Sandveld on Waterberg sandstone | Clarens sandstone hills with *Terminalia* *sericea* bush savanna or Soutpansberg group mountains with *Burkea* *africana* tree savanna |

^*^Landscapes are defined as “An area with a specific geomorphology, macroclimate, soil and vegetation pattern, and associated fauna” (Gertenbach 1983).

Reference

Gertenbach, W. P. D. 1983. Landscapes of the Kruger National Park. Koedoe 26:9–121.
