# Supplementary material for: Protected-Area Boundaries as Filters of Plant Invasions
Source: Conserv Biol. 2011 Apr;25(2):400–5. doi: 10.1111/j.1523-1739.2010.01617.x (PMC3085078; doi:10.1111/j.1523-1739.2010.01617.x)
Supplement: Supplementary file 3 [file cobi0025-0400-SD3.doc]

**Supporting Information**

**Appendix S3. D**etails on statistical analyses

The numbers of alien records could be biased by sampling intensity, for example, the park staff might have been collecting records in the surroundings of the boundary more intensively than in park interior. To test if this was the case, the numbers and proportions of alien records were compared for their trends with increasing distance from park boundary up to 1700 m, a distance for which both measures were available, by using curvilinear polynomial regression (Sokal & Rohlf 1995). Before this analysis, numbers were square rooted and proportions angular transformed to normalize the data (Sokal & Rohlf 1995) and standardized to zero mean and variance one, separately for each monitoring method, to make the two measures comparable. Trends were then measured by ANCOVA with monitoring method as a two level factor and powers of distant classes as covariates. The powers were added sequentially until the addition caused insignificant reduction in explained variance. The similarity between trends was then assessed by testing significance of interaction between monitoring method and distance classes in deletion tests. Explained variance of individual models was compared based on r^2^_adj_, which takes into account number of model parameters (e.g. Quinn & Keough 2002).

To reveal if there was a threshold distance from which the proportions of alien records level off, we assessed a region of effective neutrality (Trexler & Travis 1993) by iterations of locally weighted scatterplot smoothing (LOWESS) regression model (Cleveland 1985; Chambers et al. 1983) on square root transformed alien numbers. The iterations differed in local sensitivity expressed as a span of smoothing between 0–1, where span = 1 corresponds to standard linear regression, with complexity described as equivalent number of parameters (ENP) in curvilinear regression (Cleveland 1993). We started with a small value of span (low smoothing and high proportion of explained variance, Trexler & Travis 1993) and increased the span slowly to the point at which the LOWESS model significantly (P<0.05) differed in ANOVA deletion test from the starting model; a slightly smaller value of span, which did not differ significantly from the starting model, was chosen for final interpretation (S-PLUS 4 1997). Residuals of all models were checked to verify whether they do not show any pattern (Cleveland 1985). Calculations were done in S-PLUS v. 8.1.1 (TIBCO Software).

The permeability of park edge to invasions was analyzed by dividing segments into those with alien species present and absent, therefore the response variable was yes/no, and environmental characteristics measured within the KNP and outside the park were explanatory variables. Proportional explanatory variables were angular transformed to normalize the data, continuous variables checked for normality and standardized to zero mean and unit variance to achieve their comparable influence. Using the standardized values, collinearity was checked by calculating tolerance values among all continuous explanatory variables. Tolerances less than 0.1 were considered as values which can prevent a reliable estimate of linear model parameters (Quinn & Keough 2002). These values appeared for 10 of the 28 examined variables outside the KNP boundary, and regularly repeated for some of the variables also when collinearity checked separately at 1, 5, 10 and 50 km radius outside the KNP boundary. Consequently, instead of generalized linear models (GLMs) which would give unreliable estimates, the question what factors influence the permeability of the edge was first examined by binary classification trees (Breiman et al. 1984), a non-parametric statistical method for which collinearity of the explanatory variables cannot prevent reliable estimates. The use of the trees appeared preferable to GLMs also because some of the explanatory variables were skewed to the left, and no transformation was able to normalize the data for their proper use in GLMs. In regression trees, non-normal distribution cannot prevent reliable estimates because the trees are invariant to monotonic transformations of explanatory variables, and the explanatory variables thus do not need any transformation before analyses (De’ath & Fabricius 2000).

The classification trees were constructed in CART v. 6.0 (Breiman et al. 1984; Steinberg & Colla 1995) by binary recursive partitioning, using the default “Gini” impurity measure as the splitting index. To determine the optimal tree, a sequence of nested trees of decreasing size, each of them being the best of all trees of its size, were constructed, and their resubstitution relative errors estimated. Ten-fold cross-validation was used to obtain estimates of cross-validated relative errors of these trees. These estimates were then plotted against tree size, and the optimal tree chosen both based on the minimum cost tree rule, which minimizes the cross validated error (the default setting in CART v 6.0; Steinberg & Colla 1995), and based on the (ii) 1–SE rule, which minimizes cross-validated error within one standard error of the minimum (Breiman et al. 1984). A series of 50 cross-validations were run, and the modal (most likely) single optimal tree chosen for description (De’ath & Fabricius 2000). Because high categorical explanatory variables have higher splitting power than continuous variables, to prevent the high categorical explanatory variable landscape (22 categories) to have inherent advantage over continuous variables, penalization rules for high category variables (Steinberg & Colla 1995) were applied.

The environmental characteristics chosen by the optimal classification tree as the best predictors of the permeability of park edge lacked collinearity and could be used as explanatory variables in logistic regression (Chytrý et al*.* 2008; Hejda et al*.* 2009; Pyšek et al*.* 2009) with binomial errors and logit link function (Quinn & Keough 2002), in which alien species presence or absence in individual segments was the response variable. Overall significance of the logistic regression and significance of individual parameters and their interaction were evaluated by deletion tests based on G^2^ statistic, i.e. as the deviance of the maximum likelihood model (Crawley 2002). An overall goodness of fit of the model was assessed with the Hosmer and Lemeshow (1989) test and a determined lack of fit removed by assessing which segments had the largest influence on the model. It was done by assessment of Δβ, the analogue of Cook’s statistic in logistic regression (Hosmer & Lemeshow 1989), for the individual segments. Segments with the largest Δβ were sorted in descending order and weighted out of the analysis one after another (Gilchrist & Green 1994; Křivánek et al. 2006) until the value of Hosmer and Lemeshow test (1989) indicated no evidence of the lack of fit. The quality of the final model was expressed as r^2^_L_, i.e. as an analogue of the explained variance of the ordinary regression model (Quinn & Keough 2002; Menard 2000) and as a percentage of correctly classified values for the presence/absence of an alien plant in a segment. To verify whether the neighboring segments do not exhibit more similar records than expected by chance (e.g. Legendre 1993; Lichstein 2002), spatial autocorrelations of the individual segments were calculated as autocorrelation coefficients (Legendre & Fortin 1989) on Pearson’s standardized residuals of the logistic regression. Insignificant coefficients of the residuals indicated no spatial autocorrelations among the segments (Rangel et al. 2006; Dormann et al*.* 2007). Calculations were done in S-PLUS v. 8.1.1 (TIBCO Software) and SPSS v. 17 (SPSS Inc.).

**References**

Bourg, N. A., W. J. McShea, and D. E. Gill. 2005. Putting a CART before the search: successful habitat prediction for a rare forest herb. Ecology **86:**2793–2804.

Breiman, L., J. H. Friedman, R. A. Olshen, and C. G. Stone. 1984. Classification and Regression Trees. Wadsworth International Group, California.

Chambers, J. M., W. S. Cleveland, B. Kleiner, and P. A. Tukey. 1983. Graphical Methods for Data Analysis. Wadsworth International Group, Belmont.

Chytrý, M., V. Jarošík, P. Pyšek, O. Hájek, I. Knollová, L. Tichý, and J. Danihelka. 2008. Separating habitat invasibility by alien plants from the actual level of invasion. Ecology **89:**1541–1553.

Cleveland, W. S. 1985. The Elements of Graphing Data. Monterey, Wadsworth.

Cleveland, W. S., E. Grosse, and W. M. Shyu. 1993. Local Regression Models. Pages 309–376 in S. J. M. Chambers, and T. J. Hastie, editors. Statistical Models in S. Chapman & Hall, New York.

Crawley, M. J. 2002. Statistical Computing: an Introduction to Data Analysis Using S-Plus*.* Wiley, New York.

De’ath, G., and K. E. Fabricius. 2000. Classification and regression trees: a powerful yet simple technique for ecological data analysis. Ecology **81:**3178–3192.

Dormann, C. F., J. M. McPherson, M. B. Araújo, R. Bivand, J. Bolliger, G. Carl, et al. 2007. Incorporating spatial autocorrelation in the analysis of ecological species distribution data: a user’s guide. Ecography **30:**609–628.

Gilchrist, R., and P. Green 1994. The theory of generalized linear models. Pages 259–305 in B. Francis, M. Green, and C. Paine, editors. The GLIM System Release 4 Manual. Clarendon Press, Oxford.

Hejda, M., P. Pyšek, and V. Jarošík. 2009. Impact of invasive plants on the species richness, diversity and composition of invaded communities. Journal of Ecology **97:**393–403.

Hosmer, D. W., and S. Lemeshow. 1989. Applied Logistic Regression. Wiley, New York.

Křivánek, M., P. Pyšek, and V. Jarošík. 2006. Planting history and propagule pressure as predictors of invasion by woody species in a temperate region. Conservation Biology **20:**1487–1498.

Legendre, P. 1993. Spatial autocorrelation: trouble or new paradigm? Ecology **74:**1659–1673.

Legendre, P., and M.-J., Fortin. 1989. Spatial pattern and ecological analysis. Vegetatio **80:**107–138.

Lichstein, J. W., T. R. Simons, S. A. Shriner, and K. E. Franzreb. 2002. Spatial autocorrelation and autoregressive models in ecology. Ecological Monographs **72:**445–463.

Menard, S. 2000. Coefficient of determination for multiple logistic regression analysis. The American Statistician **54:**17–24.

Pyšek, P., M. Křivánek, and V. Jarošík. 2009. Planting intensity, residence time, and species traits determine invasion success of alien woody species. Ecology **90:**2734–2744.

Quinn, G. P., and M. J. Keough. 2002. Experimental Design and Data Analysis for Biologists. Cambridge University Press, Cambridge.

Rangel, T.F.L.V.B., J. A. F. Diniz-Filho, and L. M. Bini. 2006. Spatial patterns of terrestrial vertebrate species richness in the Brazilian Cerrado. Global Ecology and Biogeography **15:**321–327.

S-PLUS 4. 1997. Guide to Statistics. MathSoft, Seattle.

Sokal, R. R., and F. J. Rohlf. 1995. Biometry. Freeman, San Francisco.

Steinberg, G., and P. Colla. 1995. CART: Tree-structured Non-parametric Data Analysis*.* Salford Systems, San Diego.

Trexler, J. C., and J. Travis. 1993. Nontraditional regression analyses. Ecology **74:**1629–1637.
