# Supplementary material for: Protected-Area Boundaries as Filters of Plant Invasions
Source: Conserv Biol. 2011 Apr;25(2):400–5. doi: 10.1111/j.1523-1739.2010.01617.x (PMC3085078; doi:10.1111/j.1523-1739.2010.01617.x)
Supplement: Supplementary file 4 [file cobi0025-0400-SD4.doc]

**Supporting Information**

**Appendix S4.** A comparison of number and proportion of presence records of non-native plants

Comparison of non-native plant record trends in 100 m distance classes from the KNP boundary for two monitoring methods; non-native numbers and their proportional representation. The trends differ significantly only in the fourth power of polynomial function (deletion test on interaction between monitoring method and polynomial function which includes the fourth power: F = 3.67; df = 4, 28; P = 0.018; r^2^_adj_ for model with the fourth power polynomial function = 0.951) but the differences are insignificant for polynomial function with only powers up to the third degree (deletion test on interaction between monitoring method and polynomial function up to the cubic power: F = 0.46; df = 3, 29; P = 0.71; r^2^_adj_ for model with the third power polynomial function = 0.936); the fourth power appeared significant for the numbers (0.99 ± 0.31 [value ± standard error]; t = 3.23; df = 24; P = 0.0035), but insignificant (-0.55 ± 0.31; t = 1.80; P = 0.084) for the proportions. The minimal adequate models with all parameters significant, where x and their powers measure the distance from the boundary, are: proportions = -3.34x +1.63x^2^ -1.05x^3^ (F = 59.95; df = 3, 13; P < 0.0001; r^2^adj = 0.917); numbers = -3.15x + 2.01x^2^ -1.14x^3^ + 0.70x^4^ (F = 233.5; df = 4, 12; P < 0.0001; r^2^adj = 0.983); intercepts are zero due to standardizations.
