# Supplementary material for: Protected-Area Boundaries as Filters of Plant Invasions
Source: Conserv Biol. 2011 Apr;25(2):400–5. doi: 10.1111/j.1523-1739.2010.01617.x (PMC3085078; doi:10.1111/j.1523-1739.2010.01617.x)
Supplement: Supplementary file 5 [file cobi0025-0400-SD5.doc]

**Supporting Information**

**Appendix S5.** The logistic model explaining the probability of non-native plant presence in boundary segments

The probability of non-native plant presence was associated with water runoff (0–27 million m^3^) and major road density within 10 km radius outside the KNP boundary (0–0.15 km/km^2^). Overall significance of the model is G^2^ = 531.54, df=3, P<0.0001. Parameters of the model are given in the Table below. ASE are asymptotic standard errors of the parameters; standardized estimates made the parameters comparable at the same scale. G^2^ tests describe significance of the parameters based on likelihood ratio χ^2^ statistics (df are degrees of freedom and P probabilities of the statistics). Overall goodness of fit based on the Hosmer and Lemeshow test (1989) χ^2^ = 9.71, df = 6, NS; overall explained variance r^2^_L_ = 0.655, percentage of correctly classified values for the presence of a non-native species in a segment 90.4, for its absence 92.0, and the overall correctly classified percentage of values 91.3. All values are given after removing 40 segments causing a lack of fit in the overall goodness of fit of the model [Hosmer and Lemeshow test (1989) for all segments: χ^2^ = 54.20, df = 6, P < 0.0001]. All autocorrelation coefficients among Pearson’s standardized residuals of the model are insignificant. This indicates that neighboring boundary segments are not spatially correlated and the explained variability of the model does not violate statistical assumption of error independence.

| Parameter | Estimate | ASE | Standardized estimate | ASE | G^2^ | df | P |
| --- | --- | --- | --- | --- | --- | --- | --- |
| Intercept | -6.30 | 0.66 | -0.73 | 0.18 |  |  |  |
| Run-off | 0.74 | 0.08 | 3.37 | 0.31 | 264.82 | 1 | < 0.0001 |
| Road density | 69.70 | 8.59 | 1.54 | 0.20 | 127.96 | 1 | < 0.0001 |
| Run-off × Road density | -5.90 | 0.89 | -1.63 | 0.25 | 49.13 | 1 | < 0.0001 |
