# Supplementary material for: Protected-Area Boundaries as Filters of Plant Invasions
Source: Conserv Biol. 2011 Apr;25(2):400–5. doi: 10.1111/j.1523-1739.2010.01617.x (PMC3085078; doi:10.1111/j.1523-1739.2010.01617.x)
Supplement: Supplementary file 6 [file cobi0025-0400-SD6.doc]

**Supporting Information**

**Appendix S6.** Alternative model to the optimal classification tree

The alternative categorical split “Run off” (none: no rivers intersected the segment; low: 2-10; medium: 10-15; high: 15-26 million m^3^ / quaternary watershed / annum) replaced the continuous primary split “Water run-off” at node 1 of the optimal tree (Fig. 3) as a surrogate with association value = 0.86. Surrogates describe splitting rules that closely mimic the action of primary splits, with the highest possible value 1.0 corresponding to the surrogate that produces exactly the same split as the primary split. Overall misclassification rate of the model is 13.5%, sensitivity 0.90 and specificity 0.80. “Natural areas outside” refers to the percentage of natural areas in a 5-km radius outside the KNP boundary, “Road present inside” refers to the presence or absence of roads in a studied segment inside KNP. Otherwise as in Fig. 3.
